# U0126 and BAY11-7082 Inhibit the Progression of Endometriosis in a Rat Model by Suppressing the MEK/ERK/NF-κB Pathway

**DOI:** 10.1089/whr.2021.0151

**Published:** 2023-02-06

**Authors:** Fang Wang, Yong Mei Li, Ru Yue Li, Yu E. Yang, Meng Wei, Chunfang Ha

**Affiliations:** ^1^ Department of Obstetrics and Gynecology, People's Hospital of Ningxia Hui Autonomous Region, Yinchuan, Ningxia, China.; ^2^ Department of Gynecology, General Hospital of Ningxia Medical University, Yinchuan, Ningxia, China.; ^3^ Department of Clinical Medicine, Ningxia Medical University, Yinchuan, Ningxia, China.

**Keywords:** endometriosis, U0126, BAY11-708, MEK/ERK/NF-κB

## Abstract

Endometriosis is an aggressive disease. It is the main cause of chronic pelvic pain, dysmenorrhea, and infertility, affecting the well-being of women. This study aimed to explore the role of U0126 and BAY11-7082 in endometriosis (EMs) treatment in rats by targeting the MEK/ERK/NF-κB pathway. The EMs model was generated and the rats were divided into model, dimethyl sulfoxide, U0126, BAY11-708, and control groups (Sham operation group). After 4 weeks of treatment, the rats were sacrificed. Compared with model group, U0126 and BAY11-7082 treatment significantly inhibited ectopic lesion growth, glandular hyperplasia, and interstitial inflammation. Compared to control group, PCNA and MMP9 levels were significantly increased in the eutopic and ectopic endometrial tissues of model group; the levels of MEK/ERK/NF-κB pathway proteins also increased significantly. Compared with model group, MEK, ERK, and NF-κB levels decreased significantly after U0126 treatment and NF-κB protein expression decreased significantly after BAY11-7082 treatment, with no significant difference in MEK and ERK levels. The proliferation and invasion activities of eutopic and ectopic endometrial cells also significantly decreased after U0126 and BAY11-7082 treatment. In summary, our results showed that U0126 and BAY11-7082 inhibited ectopic lesion growth, glandular hyperplasia, and interstitial inflammatory response in EMs rats by inhibiting the MEK/ERK/NF-κB signaling pathway.

## Introduction

Endometriosis (EMs) is a chronic inflammatory and hormone-dependent disease, which is defined as the functionally active ectopic growth and survival of endometrial tissue outside the uterine cavity and the formation of a cystic structure.^1^ It is a progressive disease causing infertility and the main clinical symptoms include chronic pelvic pain, dysmenorrhea, and menstrual disorders. Its prevalence in women of childbearing age is 25%–50%, of which 30%–50% become infertile. In recent years, the age of onset of EMs has become earlier than those previously reported.^2,3^ Studies have shown that EMs is an endometrial abnormal genetic disease caused by increased endometrial cell activity, which is related to the *ex situ* adhesion, proliferation, and invasiveness of endometrial cells.^4,5^

To date, the pathophysiological mechanism of EMs is still based on the theory of blood flow, which lacks evidence of its development and progression, suggesting that other factors are involved in the formation of EMs.^6,7^ Studies have shown that the pathophysiological processes of chronic inflammatory reactions, cell proliferation, and cell invasion are closely related to the development and recurrence of Ems.^8^ The current treatment of the disease is mainly surgery and medication and laparoscopy is still the gold standard for its diagnosis. Studies have shown that the pathogenesis of the disease involves multiple genetic inheritances, immune abnormalities, environmental factors, abnormal angiogenesis, abnormal endometrial cell differentiation, and other factors.^9–11^ In addition to the side effects, the success rate of treatment is usually disappointing. Therefore, there is an urgent need for effective treatment options.

The MAPK/ERK mitogen-activated protein kinase cascade is a key intracellular signal transduction pathway that regulates a variety of biological functions. These include cell proliferation, survival, apoptosis, angiogenesis, migration, and invasion.^12–15^ The activation of MAPK/ERK promotes inflammatory transformation, thereby activating tumor necrosis factor α (TNFα) or the downstream target NF-κB.^16^ A previous study indicated that lidocaine inhibited the activation of the MAPK/ERK-NF-κB pathway, thereby inhibiting the inflammatory response and alleviating inflammatory pain.^17^ Nuclear transcription factor κB (NF-κB), which is one of the downstream kinases of the MAPK/ERK pathway, is activated in various diseases related to tumorigenesis and is an important target in EMs.^18–20^

In addition to being closely related to inflammatory pain, the MAPK/ERK/NF-κB pathway is abnormally activated in EMs; therefore, inhibiting the MAPK/ERK pathway may be a viable option for the treatment of Ems.^21–23^ A previous study has suggested that the MAPK pathway is hyperactivated in EMs. Another study has shown that miR-196a regulates MEK/ERK signaling and mediates the aberrant expression of PGR in eutopic endometrium, at least partly hindering decidualization in Ems.^24^ Therefore, the MEK/ERK signaling pathway and NF-κB may be key targets for the treatment of EMs.

In this study, we first established a rat model of EMs based on a preliminary modeling method. We then used U0126 and BAY11-7082, an MEK and NF-κB inhibitor, to inhibit the MAPK/ERK/NF-κB signaling pathway. We recorded and observed the volume changes of ectopic lesions in each group. We performed hematoxylin and eosin (H&E) staining to observe histopathological changes, and Western blotting and immunohistochemistry (IHC) to detect the expression of MEK, ERK, NF-κB, and proliferation and invasion proteins, MMP9 and PCNA.

## Materials and Methods

### Animals

Fifty mature female Sprague-Dawley rats (8 weeks old and weighing 200 ± 20 g) were purchased from the Ningxia Medical University Experimental Animal Center (Number of Animal Use Permit: SYXK (Ning) 2015-0001). The rats were housed under conditions of 12-h light/12-h dark cycle at 22°C ± 2°C in groups of three rats per cage; rats were allowed food and water *ad libitum*. All experiments were approved by the Institutional Animal Ethics Committee of the Ningxia Medical University Experimental Animal Center.

### Induction of experimental EMs

All operations were performed under sterile and aseptic conditions. A total of 10 out of 50 female rats were randomly chosen as the sham group and the other 40 rats were established by autologous transplantation to develop the EMs model according to the Vernon and Wilson method with minor modifications. The rats were fed adaptively for 1 week. Modeling: all animals were anaesthetized with an intraperitoneal injection of 1% pentobarbital sodium (35 mg/kg).

Abdominal skin preparation, iodophor disinfection of the abdominal skin, ligation of the left uterus about 1 cm away from the ovary, and uterine horn bifurcation were performed. Next, the endometrium was cut into four pieces of 5 × 5 mm in size, and the endometrial surface was attached to the abdominal wall. The rabbits were sutured with 6-0 absorbable sutures at the four corners of the left and right abdominal walls, while in the control group, only the abdomen was opened and intimal transplantation was not allowed. Four weeks after the first surgery, a second laparotomy was performed to test the success of the model.

### Drug administration and experimental groups

U0126 (MEK1/2 inhibitor) and BAY11-7082 (NF-κB inhibitor) were dissolved in dimethyl sulfoxide (DMSO) (final concentration <1%) as stock solutions. The stock solution was diluted with phosphate-buffered saline (PBS).

All rats were randomly divided into five groups of six rats each; group 1: Sham group, rats were given sham operation and intragastrically administered the same volume of 0.9% NaCl solution, *n* = 6; group 2: Endo group, rats with autologous uterine tissue transplantation were intragastrically administered the same volume of 0.9% NaCl solution, *n* = 6; group 3: Endo+DMSO group, EMs rats, which were intraperitoneally injected with vehicle treatment (5% DMSO in 300 μL sterile PBS), *n* = 6; group 4: Endo+U0126 group, EMs rats, which were intraperitoneally injected with U0126 (20 mg/kg) in vehicle, *n* = 6; and group 5: Endo+BAY11-7082 group, EMs rats, which were intraperitoneally injected with BAY11-7082 (20 mg/kg) in vehicle, *n* = 6; all treatments were continued for 4 weeks.

### Volume measurement of ectopic endometrial lesions

After 4 weeks of treatment, all rats were sacrificed and the volumes of endometriotic foci were calculated. The volume of ectopic endometrial grafts was measured using a Vernier caliper and the spherical volume of each focus was calculated using the conventional prolate ellipsoid formula: V (mm3) = 0.524 × W × L × T, where W = width, L = length, and T = thickness (all in millimeters).

### Collection and disposal of specimens

All animals were anaesthetized and euthanized by an intraperitoneal injection of 1% pentobarbital sodium. The eutopic and ectopic tissue were obtained from each rat of the EMs group and then the tissues were trimmed into tissue blocks of about 0.5 × 0.5 × 0.5 cm, some of which were fixed with 4% paraformaldehyde for 48 hours and then cut into paraffin sections. Some sections were cooled in a liquid nitrogen tank and transferred to a refrigerator at −80℃ for subsequent Western blot analysis.

### Histopathological examination

After sacrificing the rats, the eutopic and ectopic endometrial tissues were removed and fixed at room temperature in 4% paraformaldehyde solution for paraffin embedding and sectioning. Paraffin-embedded sections were cut at 5 μm thickness and deparaffinized after dewaxing with water. Next, hematoxylin staining was performed, which follows a basic procedure: differentiation, bluing, eosin addition, gradient alcohol dehydration, and xylene solution addition. Then, after neutral gum sealing, histopathological changes of the intima were observed under the microscope.

### Immunohistochemistry

The sections were cut into 5 μm slices and deparaffinized in xylene. The next steps were as follows: dewaxing, gradient alcohol rehydration, microwave antigen repair using sodium citrate buffer, inactivation of endogenous peroxidase, and blocking using goat serum. Next, the tissues were incubated with the antibodies MEK_1/2_ (1:500, ab178876; Abcam), ERK_1/2_ (1:200, ab17942; Abcam), NF-κBp65 (1:1,00, ab86299; Abcam), PCNA (1:200, 24036-1-AP; Proteintech), and MMP9 (1:200, 27306-1-AP; Proteintech) at 4℃ overnight. The tissues were incubated at room temperature for 60 minutes, with the addition of a polymeric horseradish peroxidase (HRP) IgG antibody against rabbit for 3, 3′-diaminobenzidine staining under the microscope. Following hematoxylin staining, gradient alcohol dehydration, xylene addition, and transparent neutral gum sealing, the expression and localization of each factor were observed under the microscope.

### Western blotting

The endometrial tissues of rats stored in liquid nitrogen were obtained and total protein was extracted. The protein concentration was determined using the bicinchoninic acid assay protein extraction and detection kit. After subjecting the samples to electrophoresis, the proteins were transferred to polyvinylidene difluoride (PVDF) membranes. The blot was incubated in a 4℃ shaker overnight with the following primary antibodies: MEK_1/2_ (1:20,000, ab178876; Abcam), ERK_1/2_ (1:1,000, ab17942; Abcam), NF-κBp65 (1:1,000, ab86299; Abcam) PCNA (1:1,000, 24036-1-AP; Proteintech), MMP9 (1:500, 27306-1-AP; Proteintech), and beta-actin (1:5,000, AF7018; Affinity). The blot was then washed and incubated with the corresponding HRP-labeled anti-rabbit secondary antibody (1 × 10,000). An appropriate amount of enhanced chemiluminescence enhancer was placed on the PVDF film and Bio-Rad was used as the imaging instrument for development exposure. The intensity of the gray value of the target band was analyzed using ImageJ software.

### Statistical analysis

GraphPad Prism 7.0 software was used for all statistical analyses. Parametric measurements were performed using one-way analysis of variance (ANOVA) tests, where nonparametric tests were performed with the Mann–Whitney *U* test. *p-*Values <0.05 were considered statistically significant. Results were expressed as the mean ± standard error of the mean.

## Results

### The effects of U0126 and BAY11-7082 on the volume, macroscopic appearance, and size of the endometrial implants

**FIG. 1.**
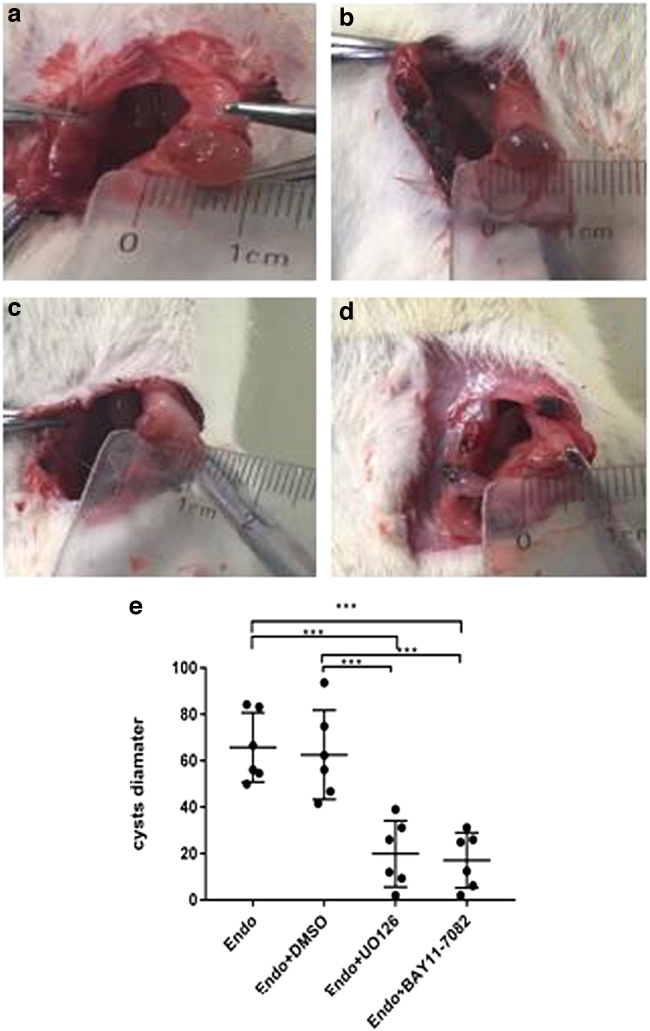
Macroscopic view of endometrial implant volumes in EMs. **(a)** Endo, **(b)** Endo+ Dmso, **(c)** Endo+U0126, **(d)** Endo+Bay 11-7082, **(f)** size of endometrial implants in the different studied groups. Values are expressed as mean ± SD (*n* = 6). Significant differences are shown as *p-value <0.05, **p-value <0.01, ***p-value <0.001, ****p-value <0.0001, *n* = 6). EMs, endometriosis; Endo, endometriosis; Endo+Bay11-7082, endometriosis+Bay11-7082; Endo+Dmso, endometriosis+Dmso; Endo+U0126, endometriosis+U0126; SD, standard deviation.

We tested whether U0126 and BAY11-7082 could be used to treat experimental EMs and inhibit the growth of endometrial implants. We examined volume changes in the grafts in each group after treatment. The macroscopic appearance and size changes in the endometrial implant volume in each group are shown in Figure 1a–f. As shown in Figure 1, compared to the model group (Endo group), the Endo+U0126 and Endo+Bay 11-7082 groups showed a significant decrease in the volume and size of the ectopic endometrial lesion (vs. model; *p* < 0.01). However, there was no significant difference in the volume and size of lesions between the model group (Endo) and DMSO group (Endo+DMSO) (vs. model; *p* > 0.05.

### The effect of U0126 and BAY11-7082 on histopathological features of ectopic endometrial lesions

**FIG. 2.**
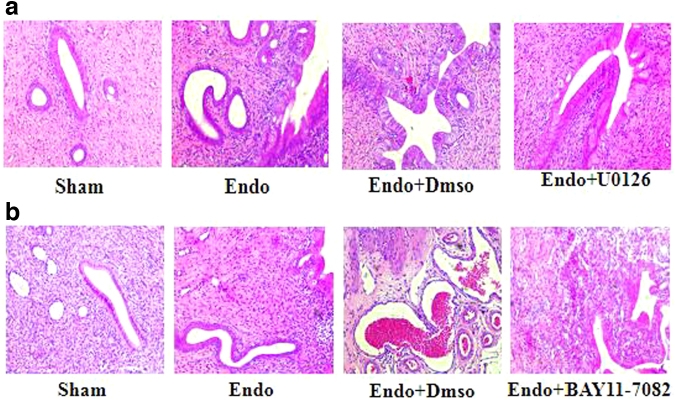
The effect of U0126 and BAY11-7082 on histopathological features of ectopic endometrial lesions **(a, b)**. Magnification, × 400 (*n* = 6) **(a)** histopathological findings in the Sham, Endo, Endo+Dmso, Endo+U0126; **(b)** histopathological findings in the Sham, Endo, Endo+Dmso, Endo+Bay11-7082.

To explore the effect of U0126 and BAY11-7082 on the histopathological changes of ectopic endometrium in experimental rats, H&E staining was performed. As is shown in Figure 2, compared with the Sham group, the ectopic lesion tissue in the model group showed multiple endometriotic gland cysts of varying sizes and interstitial reactions, with increased inflammatory cell infiltration, bleeding, and neovascularization around the glands. Compared with the model group, it can be seen that the endometrial glands reduced in size, the glandular cavity was smaller, the glandular epithelium was thinner, and the interstitial reaction was reduced in the Endo+U0126 group (Fig. 2a). Compared to the Endo and Endo+DMSO groups, incomplete endometrial epithelial and glandular cavities with interstitial reactions were observed with a significant decrease in the population of inflammatory cells in the Endo+BAY11-7082 group (Fig. 2b).

### The effect of U0126 on the expression levels of proliferation- and invasion-related factors in the eutopic and ectopic endometrial rat tissues

To investigate the potential mechanism of action of U0126 in endometrial tissues, the protein expression levels of PCNA and MMP9 were assessed by Western blotting and IHC.

**FIG. 3.**
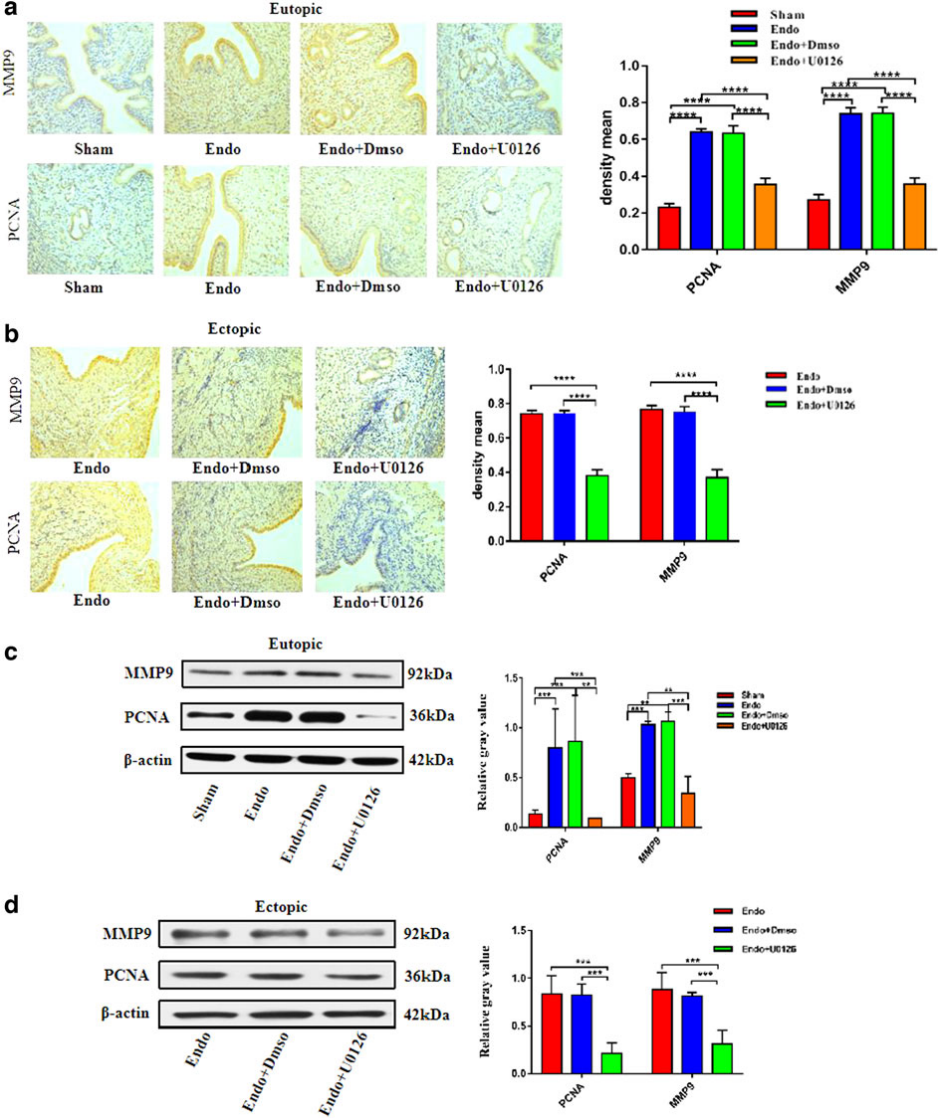
The effect of U0126 on the expression levels of proliferation and invasion relative factors (PCNA and MMP9) in the eutopic and ectopic endometrial rat tissues. Magnification, × 200 (*n* = 6) **(a, b)** IHC analysis of PCNA and MMP9 protein level in the eutopic and ectopic endometrial tissue of the Sham, Endo, Endo+Dmso, Endo+U0126); **(c, d)** Western blot analysis of PCNA and MMP9 protein level in the eutopic and ectopic endometrial tissue of the Sham, Endo, Endo+Dmso, Endo+U0126 (*p-value <0.05, **p-value <0.01, ***p-value <0.001, ****p-value <0.0001, *n* = 6). IHC, immunohistochemistry.

As is shown in Figure 3, compared with the Sham group, the expression levels of PCNA and MMP9 proteins in the eutopic and ectopic endometrium groups of the model and DMSO groups were significantly increased (vs. Sham; *p* < 0.05), respectively; after treatment with U0126, PCNA and MMP9 protein expression levels in the eutopic and ectopic EMs lesions were markedly reduced in the Endo+U0126 treatment group (vs. model; *p* < 0.05) (Fig. 3a–d). However, when compared with the model group, the expression levels of PCNA and MMP9 proteins in the eutopic and ectopic endometrial tissues of the DMSO group were not significantly different, indicating that the role of DMSO as a drug solvent had no significant effect on the proliferation and invasion of EMs (vs. model; *p* > 0.05).

### The effect of BAY11-7082 on the expression levels of proliferation- and invasion-related factors in the eutopic and ectopic endometrial rat tissues (PCNA and MMP9)

**FIG. 4.**
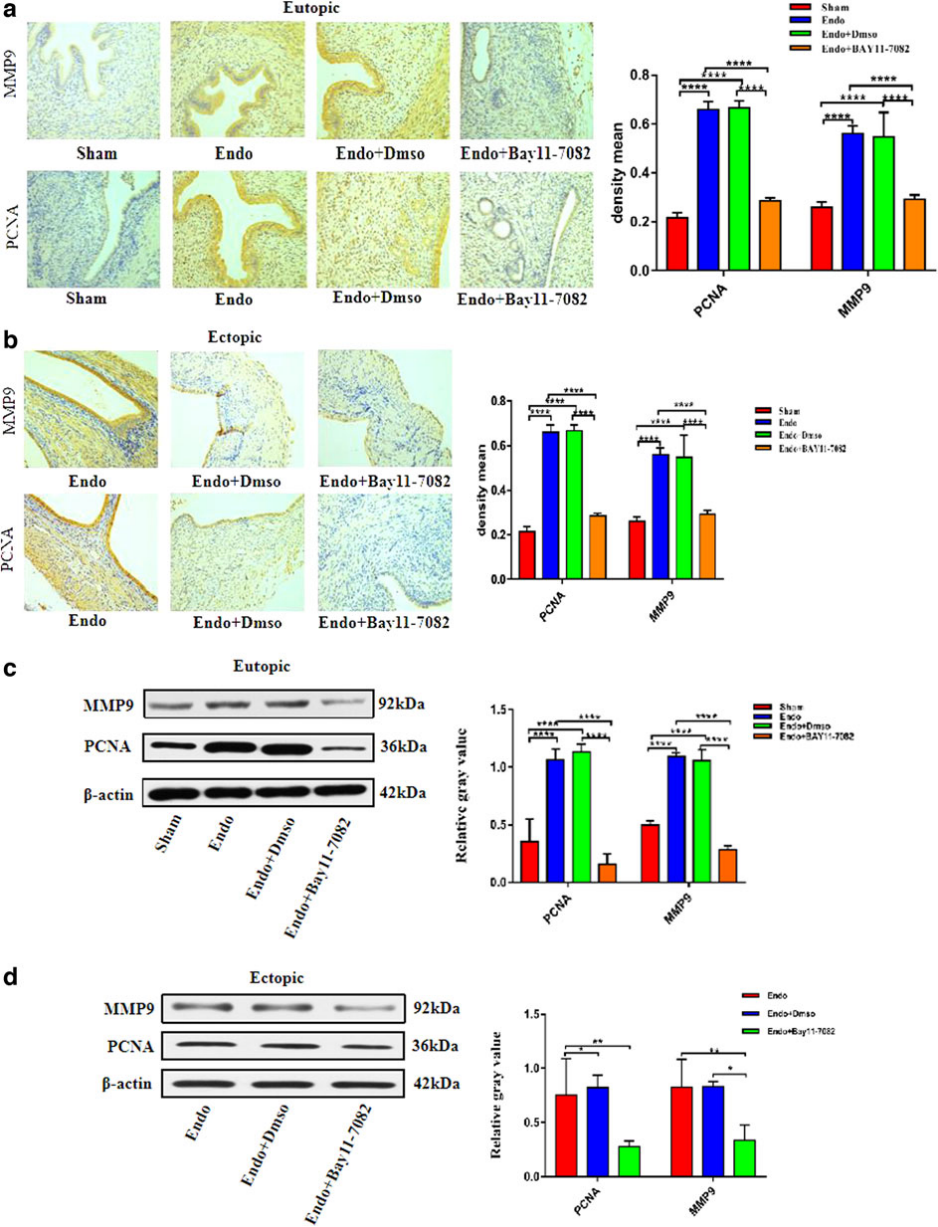
The effect of BAY11-7082 on the expression levels of proliferation and invasion relative factors (PCNA and MMP9) in the eutopic and ectopic endometrial rats tissues. Magnification, × 200 (*n* = 6) **(a, b)** IHC analysis of PCNA and MMP9 protein level in the eutopic and ectopic endometrial tissue of the Sham, Endo, Endo+Dmso, Endo+Bay11-7082; **(c, d)** Western blot analysis of PCNA and MMP9 protein level in the eutopic and ectopic endometrial tissue of the Sham, Endo, Endo+Dmso, Endo+Bay11-7082(*p-value <0.05, **p-value <0.01, ***p-value <0.001, ****p-value <0.0001, *n* = 6).

To investigate the potential mechanism of BAY11-7082 in endometrial tissues, we assessed the expression levels of proliferation and invasion proteins, PCNA and MMP9, by Western blotting and IHC. As shown in Figure 4, compared with the Sham group, the expression levels of PCNA and MMP9 protein in the eutopic and ectopic endometrium groups of the model and DMSO groups were significantly increased (vs. Sham; *p* < 0.05); after treatment with BAY11-7082, PCNA and MMP9 protein expression levels in eutopic and ectopic EMs lesions were clearly decreased in the Endo+BAY11-7082 treatment group (vs. model; *p* < 0.05) (Fig. 4a–d). However, there was no obvious difference in the protein expression levels of PCNA and MMP9 between the Endo and Endo+DMSO groups (vs. model; *p* > 0.05).

### The effect of U0126 administration on MEK1/2, ERK1/2, and NF-κBp65 expression levels in the eutopic and ectopic endometrial tissues

**FIG. 5.**
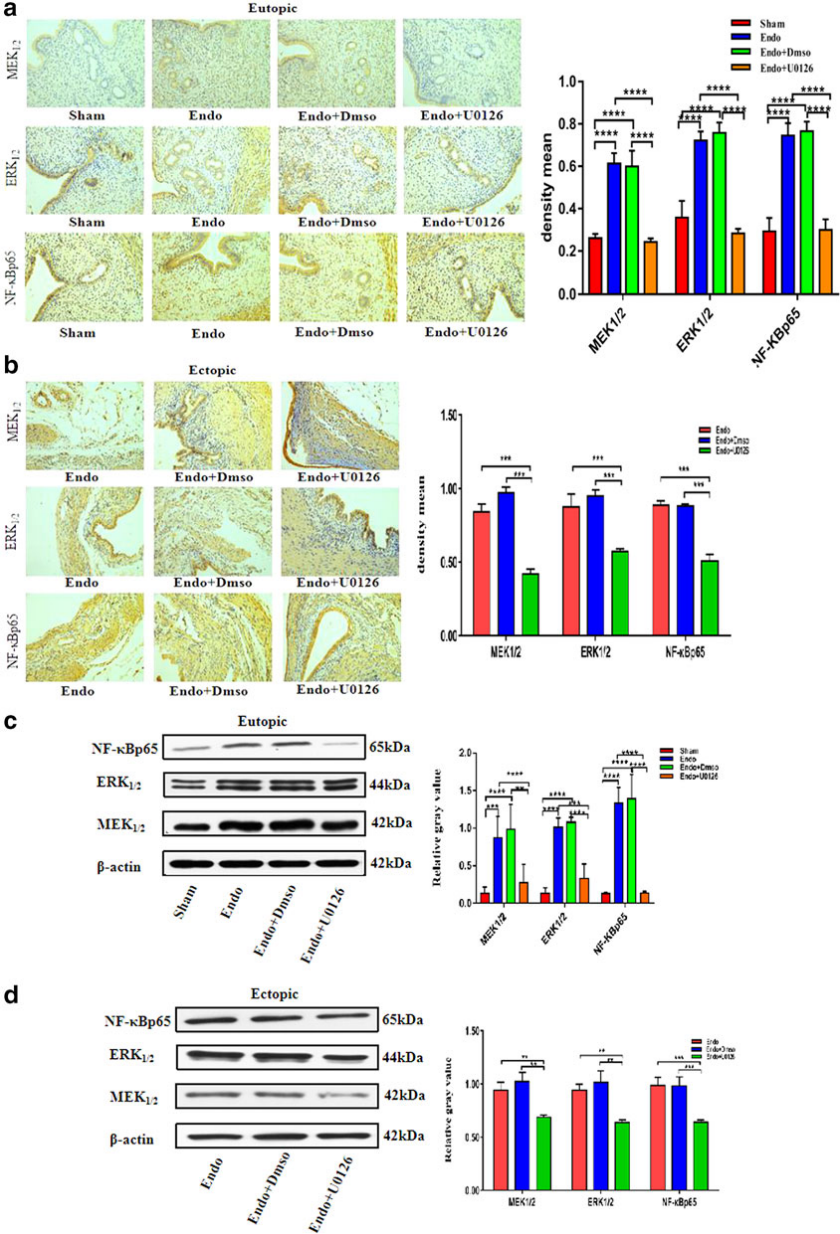
The effect of U0126 administration on MEK1/2, ERK1/2, and NF-κBp65 expression levels in the eutopic and ectopic endometrial tissues. Magnification, × 200 (*n* = 6) **(a, b)** IHC and Western blot analysis of MEK1/2, ERK1/2, and NF-κBp65 protein level in the eutopic and ectopic endometrial tissue of the Sham, Endo, Endo+Dmso, Endo+U0126; **(c, d)** Western blot analysis of MEK1/2, ERK1/2, and NF-κBp65 protein level in the eutopic and ectopic endometrial tissue of the Sham, Endo, Endo+Dmso, Endo+U0126. (*p-value <0.05, **p-value <0.01, ***p-value <0.001, ****p-value <0.0001, *n* = 6).

To investigate the potential mechanism of action of U0126 in EMs tissues, the protein expression levels of MEK1/2, ERK1/2, and NF-κBp65 were assessed by Western blotting and IHC. As is shown in Figure 5, compared with the Sham group, the expression levels of MEK/ERK and NF-κB protein in the eutopic and ectopic endometrial tissues of the model and DMSO groups were significantly increased (vs. Sham; *p* < 0.05); after treatment with U0126, MEK1/2, ERK1/2, and NF-κBp65, protein expression levels in eutopic and ectopic EMs lesions were markedly reduced in the Endo+U0126 treatment group (vs. model; *p* < 0.05) (Fig. 5a–d). Compared with the model group, there was no obvious difference in the protein expression levels of MEK1/2, ERK1/2, and NF-κBp65 in the Endo+DMSO groups (vs. model; *p* > 0.05).

### The effect of BAY11-7082 administration on MEK1/2, ERK1/2, and NF-κBp65 expression levels in the eutopic and ectopic endometrial tissues

**FIG. 6.**
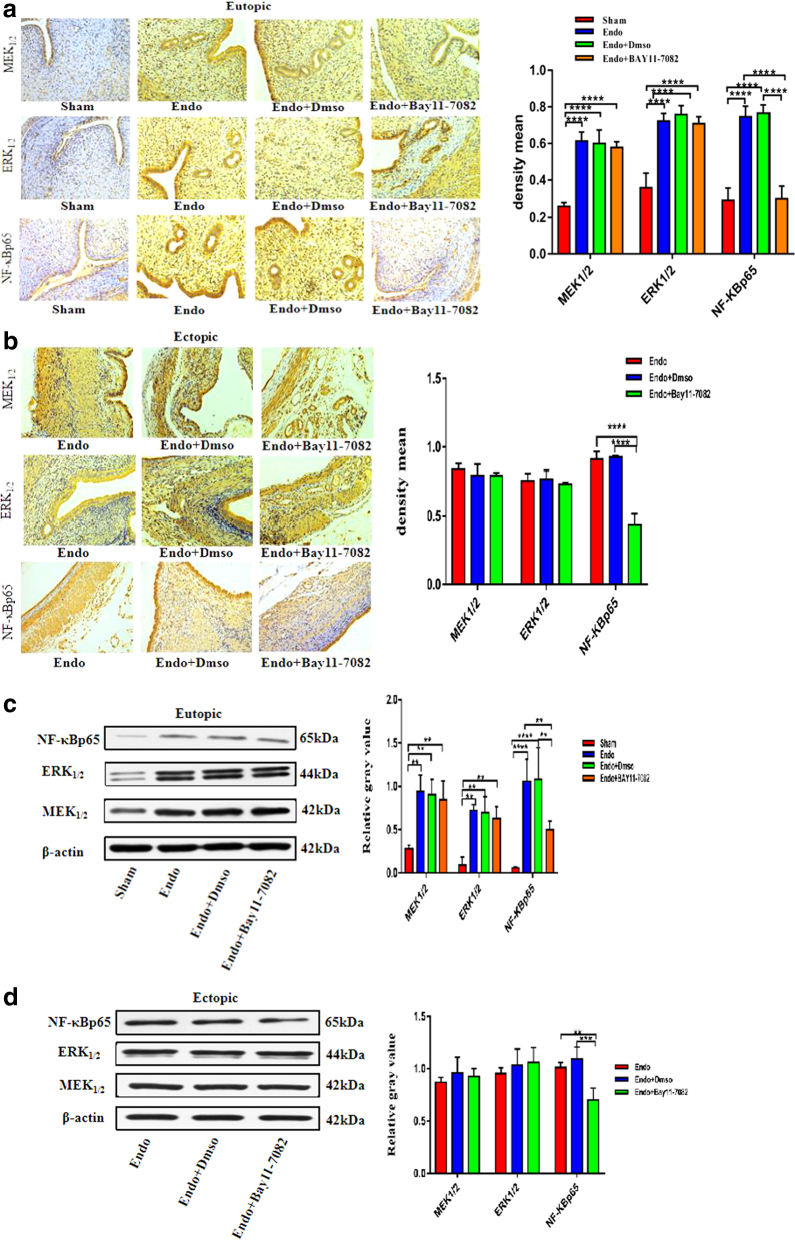
The effect of BAY11-7082 administration on MEK1/2, ERK1/2, and NF-κBp65 expression levels in the eutopic and ectopic endometrial tissues. Magnification, × 200 (*n* = 6) **(a, b)** IHC and Western blot analysis of MEK1/2, ERK1/2, and NF-κBp65 protein level in the eutopic and ectopic endometrial tissue of the Sham, Endo, Endo+Dmso, Endo+Bay11-7082; **(c, d)** Western blot analysis of MEK1/2, ERK1/2, and NF-κBp65 protein level in the eutopic and ectopic endometrial tissue of the Sham, Endo, Endo+Dmso, Endo+Bay11-7082. (*p-value <0.05, **p-value <0.01, ***p-value <0.001, ****p-value <0.0001, *n* = 6).

To investigate the potential mechanism of BAY11-7082 in EMs tissue, the protein expression levels of MEK1/2, ERK1/2, and NF-κBp65 were assessed using Western blotting and IHC. As shown in Figure 6, compared with the Sham group, the expression levels of MEK/ERK and NF-κB protein in the eutopic and ectopic endometrial tissues of the model and DMSO groups were significantly increased (vs. Sham; *p* < 0.05) after treatment with BAY11-7082.

We also tested the NF-κBp65 protein expression levels in eutopic and ectopic endometrial lesions, which were obviously decreased in the Endo+BAY11-7082 treatment group (vs. model; *p* < 0.05). However, MEK1/2 and ERK1/2 protein expression levels in eutopic and ectopic EMs lesions were not significantly different in the Endo+BAY11-7082 treatment group (vs. model; *p* > 0.05) (Fig. 6a–d). There was also no obvious difference in the protein expression levels of MEK1/2, ERK1/2, and NF-κBp65 between the Endo and Endo+DMSO groups (vs. model; *p* > 0.05).

## Discussion

To date, the exact pathophysiology of the development and progression of EMs has not been established yet. Evidence suggests that EMs is a combination of local inflammatory factor and neurogenic mediator accumulation, steroid hormone imbalance, iron and oxidative stress, and other factors.^25^ EMs has biological behaviors similar to malignant tumors, such as explantation, proliferation, invasion, and adhesion. The high incidence of EMs, various side effects of hormone administration, and high postoperative recurrence limit the clinical application of EMs drugs and surgery.^26–28^

Recent studies have found that after continuous stimulation by intracellular and extracellular factors, the MAPK/ERK pathway is over activated and the activated ERK activates many downstream effector molecules, such as NF-κB, RSK, and others, which can cause proliferation, survival, and angiogenesis of tumor cells and the transcription of different genes related to metastasis.^29–31^

These findings demonstrated that the MEK/ERK pathway that relies on MAPK kinase acts as a sensitizer of endometrial cell proliferation and upregulates the expression of NF-κB, which promotes the proliferation and invasion of endometrial stromal cells, revealing that the MEK/ERK pathway is a potential target for the treatment of EMs. MEK/ERK inhibitors may become a new type of drug for the treatment of Ems.^32–34^ Simon et al^35^ found in oral cancer research that the MEK/ERK pathway is activated in oral cancer cells.

U0126 can inhibit the activity of MEK/ERK by inhibiting the expression of MMP9 to reduce its invasiveness, revealing that MMP9 acts through the MEK/ERK signaling pathway, which promotes the invasion and metastasis of oral cancer cells; therefore, inhibiting this signaling pathway can reduce tumor invasion and metastasis; thus, it is considered that inhibiting the activation of this pathway is a new target in tumor therapy. A study on diabetes found that the MAPK/NF-κB pathway is abnormally activated; however, U0126 inhibits the MAPK/NF-κB pathway and has a protective effect on pancreatic β cells, providing a new treatment strategy for the treatment of diabetes.^36–38^ Abnormal activation of the ectopic endometrium is closely related to ectopic endometrial proliferation and aggressive enhancement of EMs.^39,40^ In EMs, U0126 and BAY11-7082 protect EMs by inhibiting the MAPK/NF-κB pathway.

To test this, we established a rat model of EMs to explore the therapeutic effect of inhibiting the MAPK/ERK/NF-κB signaling pathway in ectopic lesions in EMs rats. Our studies have confirmed that MEK_1/2_, ERK_1/2,_ and NF-κBp65 proteins are highly expressed in eutopic and ectopic tissues of EMs rats and are closely related to the enhancement of ectopic cell proliferation and invasion. By using U0126 to regulate the status of the signaling pathway, we proved that inhibiting the MAPK/ERK/NF-κB signaling pathway reduces the expression of MEK_1/2,_ ERK_1/2,_ and NF-κBp65, thereby inhibiting the proliferation and invasion of ectopic lesions so as to achieve the purpose of treating EMs.

In this model, the EMs rat model was successfully established and we found that the volume of ectopic lesions in the model group was significantly increased and abundant blood vessel formation and adhesions were observed on the surface and surrounding tissues. The volume of ectopic lesions in the U0126 and BAY11-7082 groups was significantly reduced and adhesion with the surrounding tissues was reduced. This indicates that MEK and NF-κB inhibitor drugs can effectively inhibit the expansion and growth of endometrial and ectopic lesions and control the progression of the disease. H&E staining showed that there was abundant neovascularization, glandular hyperplasia, and gland cavities in the ectopic lesion tissues of the model group.

We examined the effect of U0126 and BAY11-7082 on the pathological morphology of ectopic lesions, the results showed that compared to the sham group, the ectopic endometrial tissue in the model group had regular irregular columnar arrangement of glandular epithelial thickening, more interstitial reactions with formation and size of endometrial glands, periglandular hemorrhage, angiogenesis and massive inflammatory cell infiltration. Compared to the model groups, the U0126 and BAY11-7082 groups both showed fewer residual endometrial glands, smaller glandular epithelium, reduced interstitial response, and decreased inflammatory cell population, indicating that U0126 and BAY11-7082 can have a good therapeutic effect on EMs by inhibiting endometrial glandular stromal hyperplasia and reducing inflammation.

We further examined the effects of U0126 and BAY11-7082 on the proliferation and invasiveness of eutopic and ectopic endometrial tissues from rats in each group. The results showed that compared with the Sham group, the expression levels of PCNA and MMP9 in the eutopic and ectopic endometrial tissues of the model and DMSO groups were significantly increased, indicating that the occurrence of EMs is related to the increased aggressiveness of ectopic endometrial cells. After treatment with U0126 and BAY11-7082, the expression levels of PCNA and MMP9 were significantly decreased.

This indicates that U0126 and BAY11-7082 can effectively inhibit the growth, proliferation, and invasion of EMs lesion tissues to achieve effective treatment of EMs. To further explore the mechanism of U0126 and BAY11-7082 in the treatment of EMs rats, the expression of key proteins MEK_1/2_, ERK_1/2,_ and NF-κBp65 in the signaling pathway was determined at the protein level. The results showed that the expression levels of MEK, ERK, and NF-κB proteins in the model and DMSO groups in the eutopic and ectopic endometrial tissues were significantly increased, indicating that the overexpression of MEK protein in EMs promotes the expression of ERK and downstream effector NF-κB, which leads to abnormal activation of the MEK/ERK and NF-κB pathways in the EMs rat model and promotes the growth of ectopic lesions, which is obviously related to the occurrence and development of EMs.

Compared with the model group, the expression levels of MEK, ERK, and NF-κB proteins in the eutopic and ectopic endometrial tissues of the U0126 group were significantly reduced, indicating that U0126, as a competitive MAPK pathway inhibitor, can inhibit the upstream ERK kinase MEK. Phosphorylation, which inhibits the activation of MEK/ERK, further blocks the transcriptional activation of its downstream effector NF-κB protein and effectively inhibits the growth, proliferation, migration, and invasion of the ectopic lesions of EMs. The expression level of NF-κB protein in the eutopic and ectopic endometrial tissues of the BAY11-7082 group was significantly reduced, whereas the expression levels of MEK and ERK proteins were not significantly different, indicating that BAY11-7082 acts as a targeted inhibitor of the NF-κB pathway.

Mainly, preventing the activation of NF-κB and blocking the nuclear transduction effect of NF-κB have a therapeutic effect on EMs, but have no obvious effect on the expression levels of the upstream genes MEK and ERK. In summary, our research results show that U0126 and BAY11-7082 can significantly inhibit the growth of ectopic lesions in EMs rats and reduce gland proliferation and interstitial inflammation in the ectopic lesions of EMs rats. In response, the mechanism of action of the two drugs may target the activation of MAPK/ERK and NF-κB pathways, inhibit the expression of proliferation and invasion proteins in EMs rats, and ultimately, effectively inhibit the growth, proliferation, migration, and invasion of ectopic lesions of EMs to achieve the purpose of treating EMs.

## Conclusions

U0126 and BAY11-7082 can effectively inhibit the growth, proliferation, and invasion of ectopic lesions in EMs rats and reduce the proliferation of glands and interstitial inflammatory responses in ectopic lesions. The mechanism may be related to the inhibition of the activity of MAPK/ERK and NF-κB pathways, which are related to the treatment of EMs.

## Abbreviations Used

ANOVA: analysis of variance
DMSO: dimethyl sulfoxide
EMs: Endometriosis
ESCs: endometrial stromal cells
H&E: hematoxylin and eosin
HRP: horseradish peroxidase
IHC: immunohistochemistry
PBS: phosphate-buffered saline
PVDF: polyvinylidene difluoride
SD: standard deviation
